# Editorial: Ocular Motor and Vestibular Deficits in Neurometabolic, Neurogenetic, and Neurodegenerative Diseases

**DOI:** 10.3389/fneur.2018.00567

**Published:** 2018-07-18

**Authors:** Alessandra Rufa, Aasef G. Shaikh

**Affiliations:** ^1^Department of Neurology, University of Siena, Siena, Italy; ^2^Daroff-Dell'Osso Ocular Motility Laboratory, Neurology Service, Louis Stokes Cleveland VA Medical Center, Cleveland, OH, United States; ^3^Department of Neurology, University Hospitals and Case Western Reserve University, Cleveland, OH, United States

**Keywords:** eye movement, balance, vestibular, cerebellum, cerebrum, brainstem

The eye movements have been studied for last two centuries; from time to time the emphasis of the ocular motor literature has changed. Most early studies were clinically focused, describing phenomenology in various syndromic entities. A century later, with advent of technology allowing us to be able to quantitatively measure ocular motor behavior, the physiological investigations of eye movements intensified. The subsequent literature emphasized objective description of various classes of eye movements. There was a substantial growth of quantitative ocular motor studies in 1960s and onwards, which directly correlated with the introduction of the field to the engineers and neuroscientists. The combination between the eye movements research and engineering resulted in birth of high-resolution technology to precisely measure ocular motor function in humans and monkeys; such technology was then utilized to provide quantitative description of physiology and generation of neural control systems models. The models not only provided detailed insight into the physiology of ocular motor control, but it also provided unprecedented explanation of the pathophysiology of ocular motor deficits. The utilization of control systems models to describe disorders of ocular motor system became increasingly attractive as neurologists and ophthalmologists were trained in ocular motor physiology and engineering of motor control systems. In 1970s through 1990s, there was a rapid growth in the literature describing the basic physiology of ocular motor control, and objective description of pathophysiology of ocular motor disorders due to dysfunction in the orbit or the brain. As a result, to date, the physiology of eye movements is the most well-understood among all motor systems and various disorders of eye movements are very well-accounted for by pathophysiological principles. The era of ocular motor physiology and pathophysiology was succeeded in the modern times by utilizing the eye movements as the objective biomarkers to understand and follow complex disease processes where immune, degenerative, and developmental disorders affects the brain. There are increasing number of studies utilizing eye movements as biomarkers of the processes affecting the cognition, behavior, and attention. Invention of the user friendly off-shelf oculography techniques have catalyzed the inclusion of eye movements as biomarkers. The simplicity of the data capture strategy with modern devices have attracted more and more investigators, including those from diverse background, to use eye movements as outcome measures. In our opinion the studies of eye movements have traversed through three era—the longest and oldest phenomenological era where the clinical ocular motor features in various disorders were descried; classic physiological era where the physiology of ocular motor control was explained and control systems neuroscience thrived; and modern era of application of eye movements as biomarkers and in studies of cognitive neuroscience. Many investigators in the modern era “view” the eye movements in different perspectives—to monitor the progression of the disease and examine the response to therapy; or to study cognitive performance and attention. This special volume of Frontiers in Neurology titled “Ocular Motor and Vestibular Deficits in Neurometabolic, Neurogenetic, and Neurodegenerative Diseases” has collection of papers covering contents that were emphasized in all three era of ocular motor history.

The ability to capture eye-movements with spatial and temporal high-resolution has brought significant advancement in studying minute eye movements, such as microsaccades. These “fixational” eye movements occurring at the rate of 1–2 Hz are critical to prevent visual fading. In addition to their importance in suppressing visual adaptation and fading, they are also implicated in attention. The direct clinical implication, in addition to understanding the pathophysiological underpinnings of the given disorder, is that these fixational eye movements allow objective surrogate markers of disease progression and therapeutic outcomes. The microsaccades can be objective biomarkers in neurodegenerative disorders such as Parkinson's disease, atypical forms of parkinsonism, and dementia; neurodevelopmental disorders such as attention-deficit hyperactivity disorder and amblyopia; or immune disorders such as multiple sclerosis. A comprehensive review by Alexander and colleagues summarizes how various disorders of the nervous system affects microsaccades (Alexander et al.). Otero-Millan and colleagues then apply the principles of computational neuroscience to describe physiological underpinning for triggering of microsaccades and saccades (Otero-Millan et al.).

Although ocular motor deficits are not symptomatic in common neurological disorders such as epilepsy and dementia; they are frequently described as surrogate markers. Colnaghi et al. describe abnormality in left-ward memory guided saccades in patients with right mesial temporal lobe epilepsy with hippocampal sclerosis. In cohort of 36 patients with young onset Alzheimer's disease (including those with posterior cortical atrophy), Pavisic et al. identified a range of subtle abnormalities in basic ocular motor function. Structural cerebral lesions as seen in stroke and traumatic brain injury not only affect the timing, velocity, and accuracy of the saccades; but also their coupling with reaching limb movements (Rizzo et al. Rizzo et al. Rizzo et al.). The same concept applies to structural loss due to degenerative or immune-mediated cerebral injury.

Common neurodegenerative disorders such as Alzheimer's disease and parkinsonism are frequently associated with increasing frequency of falls and navigational impairments. Although brainstem generated vestibulo-ocular reflex are normal in these patients, it is likely that there is impairment in “cognitive” aspect of the vestibular function—especially the vestibular motion perception. Latter requires optimal interaction between vestibular and visual modalities for motion perception, and such behavior is dependent on the cortical neural substrate in temporal and parieto-temporal lobe. Cronin and colleagues comprehensively review the motion perception and non-ocular motor vestibular deficits in neurodegenerative disorders (Cronin et al.). Kheradmand and Arial report the updates on the physiology of perception of verticality and role of the cerebral cortex on such perceptual behavior (Kheradmand and Winnick). There is a desperate need to translate these physiological concepts in defining novel therapeutic strategies to restore balance function in neurodegenerative disorders.

This volume also includes novel applications of ocular motor experiments in understanding of classic degenerative, developmental, or immune disorders of the nervous system. Pretegiani and Optican confirmed that voluntary saccades are abnormal in idiopathic Parkinson's disease at all stages, but in its more severe forms both voluntary and reflexive saccades are affected. It was also shown that eye movements in Parkinson's disease is distinct from its genetic variants (Pretegiani and Optican). These differences, along with specific genetic mechanism can provide insight into the pathophysiology of various genetic forms of parkinsonian syndromes. Ghasia and Shaikh reported detailed kinematic properties of slow saccades and abnormal gaze-holding function in rare yet classic disorder of the central nervous system—the Whipple's disease (Shaikh and Ghasia). The unique combination of reported ocular motor phenomenology suggest multi-system involvement in Whipple's disease affecting the basal ganglia, brainstem, and cerebellum (Shaikh and Ghasia). Kang and colleagues have reviewed the details of dysconjugate eye movements and strabismus in parkinsonian syndromes (Kang et al.). Deep brain stimulation is successful therapy and is considered the standard of care for the treatment of Parkinson's disease. The literature on how the deep brain stimulation improves motor function is murky. Shaikh et al. reviewed physiological influence of neuromodulation with deep brain stimulation on ocular motor function in Parkinson's disease. Future studies of deep brain stimulation on eye movements, along with implementation of functional tissue activation models will provide valuable insights how the deep brain stimulation affects the motor control. Blume et al. reported disorders of saccades in rare condition called Gaucher's type 3 disease, while Federighi et al. compared ocular motor function in extremely rare genetic neurodegenerative condition called ataxia-telangiectasia *like* disorder. The syndrome of ocular palatal tremor is an acquired neurodegenerative condition characterized by quasi-sinusoidal oscillations of the eyes and palate. Tilikete and Desestret has provided an excellent review of contemporary neurology of ocular palatal tremor. Common and debilitating immune disorder, multiple sclerosis, frequently presents with disabling ocular motor deficits. Latter is frequently a diagnostic hallmark of multiple sclerosis, but it also provides a valuable marker to assess therapeutic response. Serra et al. provide an excellent outline of the ocular motor function in multiple sclerosis. Infantile nystagmus syndrome is a common childhood onset disorder of ocular motor system. Lin et al. hypothesized that spontaneous nystagmus in dark in patients with infantile nystagmus syndrome may be attributable to sensory adaptation in the optokinetic system after a sustained period of spontaneous nystagmus with directional visual input in light. An excellent review of eyelid motor control in neurodegenerative disorders further extends the scope of this Frontiers topic to another motor system that is closely related to the eye movement control (Hamedani and Gold).

The growth of studies on biomarker and behavioral and cognitive neuroscience is attributed to technical advances and availability of non-invasive cost-effective eye trackers. At the onset of quantitative oculography era, the eye movement research required a technically daunting infrastructure and highly specialized researchers to conduct the experiment. Instead the new technology is available off-shelf and is ready to use. Analysis is performed in preconfigured software that comes with the data capturing equipment. Such user-friendliness has attracted researchers who do not have expertise in ocular motor physiology, and therefore increased the application of eye movements in the fields outside of motor neuroscience. We advise a caution in interpreting the readily available data. The modern oculography techniques are not free of technical (e.g., signal noise) or biological (e.g., vestibular eye movements when head is not adequately stabilized while measurement of gaze holding) artifacts. Presence of such artifacts can be misleading leading to wrong scientific conclusions. Kaski and Bronstein have thoroughly reviewed the nature of biological artifacts in patients with Parkinson's disease.

In summary the study of ocular motility is near and dear to the hearts of many quantitative minded clinical and basic neuroscientists. It has attracted brilliant scientists from various disciplines, and such multidisciplinary mission has highly advanced the understanding of motor physiology in unequivocal fashion. Introduction of the modern, cost-effective, non-invasive, and high-resolution technology has fostered the growth of our field in various directions including computational neuroscience, neurophysiology, clinical neurology and biomarker development, and cognitive neurology. We are hopeful that collection of papers under this Frontiers Topic will be instrumental in attracting more contributors to our field.

## Author contributions

AS conceptualized the manuscript and prepared it. AR conceptualized the manuscript and edited it.

### Conflict of interest statement

The authors declare that the research was conducted in the absence of any commercial or financial relationships that could be construed as a potential conflict of interest.

